# The Story of the Insane from Year to Year

**Published:** 1895-02-02

**Authors:** 


### THE STORY OF THE INSANE FROM YEAR TO YEAR.

(7TH SERIES.)

### HEREFORD COUNTY ASYLUM.

During the year 1893 the numbers rose from 386 to 394. There were 70 admissions, 21 recoveries, and 19 deaths; and these numbers show that 30 per cent. on the admissions recovered, 4·8 died on the average number resident. We learn from Table X. that 24 of the admissions owed their insanity to hereditary influence, 7 to drink, and 4 to domestic trouble. Seven of the deaths were caused by some form of brain disease, and 2 by consumption. Dr. Chapman's report is a long and interesting one. He goes fully into the numbers of patients in his asylum in relation to the population of the county; to the average age of his patients and the death-rate among them, and he sums up as follows: "Hereford has the largest proportion of its people in the asylum of any county; but the asylum population has a mortality more resembling that of the general population than of an asylum population.

. . . Whatever circumstances may tend to produce these figures, a fair share in their production may be attributed to the hygienic efficiency of the asylum, and may be taken as evidence that everything special in the treatment or management of the patients, in which it differs from other asylums, is a difference to be commended for its success." Dr. Chapman is unquestionably right when he says that the practice of getting every patient out of bed every day is indefensible; but surely he is not correct in saying that this is "almost the rule" in many asylums—at least we hope not. If it exist at all it must be a survival of some well-nigh forgotten custom of bygone days; and he is equally right when he says that the "bed system" is better than chemical restraint, or placing patients wedged together, like sardines in a box, behind heavy tables; but we would ask where is this "wedging" to be seen? Either our experience has been exceptional, or Dr. Chapman is wrong in his illustration. We notice that a system of warming is being introduced, and if this be one of those systems where the patients are compelled to breathe unduly warmed air, Dr. Chapman must look out for an increase in his death-rate from phthisis, more especially if open fires be not used in addition to the "system." The committee report favourably of Dr. Chapman's management of the asylum during a trying year, and they attribute to his "foresight and care" the fact that the patients did not suffer while the works were in progress. The average weekly cost of maintenance is a fraction under 9s. 1d. per head.

SOMERSET COUNTY ASYLUM.

Here the numbers were almost stationary, being 867 on January 1st, and 865 on December 31st. There were 210 admissions, 103 recoveries, and 73 deaths. The percentage of recoveries was 53·55 on the admissions and the deaths 8·49 on the average number resident. Of the year's admissions, 21 owed their insanity to hereditary influence, 19 to drink, and 12 to domestic trouble. Cerebral and spinal diseases account for 27 of the deaths, consumption for 11, diarrhœa for 2, and enteric fever for 2. Regarding the latter, Dr. Wade says: "We were not able to throw any light on the cause of these cases, which occurred in different wards. No sanitary defect could be discovered in the wards in question. The disease did not spread beyond the patients first attacked." The asylum is full, and 30 patients are boarded out at the Bristol Asylum, and there are already a total of 115 at Gloucester, Exeter, and Plymouth Asylums. The committee say that "Dr. Wade devotes himself to promote the efficiency of the asylum, and to the care of those under his charge." The weekly charge to the unions is 9s. 4d. per head.

